# Dialyzed Iron

**Published:** 1879-05

**Authors:** 


A contribution to the Hæmatinic properties of Dialyzed Iron, by Robert Amory, M. D., of Longwood, Mass., amounts to a demonstration of the Hæmatinic properties of Iron, and has attracted much attention. He writes to Wyeth & Brother as follows:

Longwood, Mass., April 9th, 1879.

Messrs. Wyeth & Bro.,

*Dear Sirs:*—In answer to your inquiry as contained in your note of yesterday, I will say that in the experiments which are detailed in the Boston Medical and Surgical Journal for April 3d, I used only the solution of Dialyzed Iron manufactured by your firm.

Yours, Respectfully,

ROB'T AMORY.

